# Left Frontal Connectivity Moderates the Relationship between Inflammation and Cognitive Performance in Patients following Cardiac Surgery: A Longitudinal fMRI Study

**DOI:** 10.34133/research.0893

**Published:** 2025-09-19

**Authors:** Qihui Wang, Xiuqin Jia, Meiyan Zhou, Jiajia Zhang, Yangzi Zhu, Daqing Ma, Liwei Wang, Qi Yang

**Affiliations:** ^1^ Department of Radiology, Beijing Chaoyang Hospital, Capital Medical University, Beijing, China.; ^2^ Key Lab of Medical Engineering for Cardiovascular Disease, Ministry of Education, Beijing, China.; ^3^ Medical Research Center, Beijing Chaoyang Hospital, Capital Medical University, Beijing, China.; ^4^ Department of Anesthesiology, Xuzhou Central Hospital, Xuzhou, China.; ^5^ Division of Anesthetics, Pain Medicine and Intensive Care, Department of Surgery and Cancer, Faculty of Medicine, Imperial College London, Chelsea and Westminster Hospital, London, UK.; ^6^ Perioperative and Systems Medicine Laboratory, The Children’s Hospital, Zhejiang University School of Medicine, National Clinical Research Centre for Child Health, Hangzhou, China.; ^7^ Department of Anesthesiology, The First Affiliated Hospital, Ningbo University, Ningbo, China.; ^8^ Laboratory for Clinical Medicine, Capital Medical University, Beijing, China.

## Abstract

**Background:** Cardiac surgery patients frequently experience perioperative neurocognitive disorder. Cognitive reserve (CR) is known to mitigate such deficits. Here, we investigate whether global left frontal cortex (gLFC) connectivity, a neural proxy of CR, modulates the relationship between postoperative inflammation and cognitive recovery. **Methods:** Twenty-five patients scheduled for heart valve replacement surgery were compared to healthy controls using neuropsychological assessments, magnetic resonance imaging, and cytokine levels measured at 3 intervals (before surgery and 7 and 30 days after surgery). Linear regression analysis was used to examine the relationship between the increase in inflammation markers on postoperative day 7 (ΔInflammatory factor_[7d-baseline]_) and changes in cognition from postoperative day 7 to 30 (ΔNeuropsychological assessment_[30d-7d]_). Moderation analysis combined with Johnson–Neyman threshold testing was performed to assess how gLFC connectivity across 3 time points moderated the relationship between ΔIL-6_[7d-baseline]_ and cognitive change. **Results:** Patients exhibited significant cognitive decline, particularly in executive and memory function, as well as decreased gLFC connectivity on postoperative day 7, compared to controls. This decline was followed by recovery on postoperative day 30. In patients, ΔIL-6_[7d-baseline]_ was negatively correlated with ΔCorsi block test_(30d-7d)_: *β* = −0.62, *P* < 0.001 and ΔDigit symbol test_(30d-7d)_: *β* = −0.47, *P* = 0.017. The interactions of gLFC connectivity at 3 time points × ΔIL-6_[7d-baseline]_ were separately significant on postoperative cognitive recovery. Johnson–Neyman analysis revealed that the effect of ΔIL-6_[7d-baseline]_ was significant when gLFC connectivity was within a specific range. **Conclusion:** These findings suggest that gLFC connectivity, reflecting CR, may serve as a target for interventions to enhance cognitive resilience in cardiac surgery patients.

## Introduction

Perioperative neurocognitive disorder (PND) is a frequent complication that occurs postoperatively, particularly in the first week following anesthesia and surgery [1,2]. It is linked to an increased risk of Alzheimer’s disease and mortality [3,4]. Early intervention to enhance cognitive recovery has shown the potential to improve long-term surgical outcomes [5,6]. Current studies demonstrated that the inflammatory response triggered by surgical procedures contributes to the occurrence of postoperative cognitive dysfunction [7,8], which may be associated with disruptions in functional connectivity [9]. However, it remains unclear whether inflammation affects cognitive recovery after cardiac surgery.

Cognitive reserve (CR) is defined as the brain’s adaptive capacity to preserve cognitive function in the presence of neuropathology, achieved through efficient network utilization or compensatory mechanisms [10,11]. CR may mitigate the adverse effects of surgical stress on cognition through compensatory or preexisting cognitive processing strategies [12,13]. Higher CR has been shown to predict better postoperative cognitive outcomes in cardiac surgery patients, likely by bolstering resilience against brain pathophysiological alterations [14].

Thus far, CR has traditionally been measured using static proxies based on environmental factors or early life experiences, such as years of education [15,16]. However, CR is subject to dynamic changes over time in response to brain aging or injury [17]. Recent studies have demonstrated that global connectivity of the left frontal cortex (gLFC connectivity) serves as a neural substrate of CR in Alzheimer’s disease [18–20]. It may also serve as an indicator of the efficiency of higher-order cognitive networks [21]. This is predicated on the hypothesis that it potentially reflects acute resilience to surgery-specific inflammatory responses and helps to bridge the gap between chronic neurodegeneration and acute surgical stress. However, whether dynamic changes in gLFC connectivity influence postoperative cognitive recovery after cardiac surgery is unknown.

We aimed to investigate (a) dynamic postoperative changes in CR through longitudinal gLFC connectivity assessments and (b) the interaction between inflammation and CR in postoperative cognitive recovery. We hypothesized that surgery-induced inflammation negatively impacts cognitive recovery and that higher preoperative and postoperative gLFC connectivity mitigates this effect.

## Results

### Demographic characteristics and neuropsychological assessment outcomes

At baseline, patients exhibited significantly higher concentrations of interleukin-6 (IL-6) and tumor necrosis factor-α (TNF-α) in comparison to healthy controls (HCs) (*P* < 0.001). However, no significant variations were observed in other demographics or clinical parameters between the 2 groups (Table 1).

**Table 1.** Comparison of demographic and clinical characteristics between healthy controls and cardiac surgery patients. Data are presented as the mean ± standard deviation, median [25th, 75th], or number (percentage).

Characteristics	Patients	Healthy controls	*P*
	(*n* = 25)	(*n* = 25)	
Demographic characteristics			
Age (years)	52.0 ± 10.7	52.1 ± 10.0	0.967
Female	14 (56%)	15 (60%)	0.774
BMI (kg/m^2^)	25.2 ± 1.5	25.1 ± 1.3	0.833
Education (years)	9 [7, 10.5]	9 [7, 11]	0.961
Clinical characteristics			
History of hypertension	4 (16%)	4 (16%)	1.000
Diabetes mellitus	3 (12%)	1 (4%)	0.297
MMSE score	26.4 ± 1.8	27.1 ± 1.8	0.143
LVEF (%)	55 [52.5, 57]	56 [54.5, 58]	0.166
Surgical characteristics			
Duration of procedure (min)	185.3 ± 29.0	/	/
Duration of pump CPB (min)	82.2 ± 20.8	/	/
Inflammatory factors IL-6 (pg ml^−1^)			
Baseline	13.8 ± 1.4	9.3 ± 1.3	< 0.001
Postoperative day 7	32.2 ± 1.8	/	/
Postoperative day 30	19.0 ± 1.6	/	/
Inflammatory factors TNF-α (pg ml^−1^)			
Baseline	9.8 ± 1.3	7.0 ± 1.3	< 0.001
Postoperative day 7	21.0 ± 2.0	/	/
Postoperative day 30	13.6 ± 1.4	/	/

BMI, body mass index; MMSE, Mini-Mental State Examination; LVEF, left ventricular ejection fraction; CPB, cardiopulmonary bypass; IL-6, interleukin-6; TNF-α, tumor necrosis factor-α

Table 2 shows a comparison of neuropsychological assessments between patients and HCs at the cross-sectional and longitudinal levels. At the cross-sectional level, statistical analysis revealed no significant baseline differences in cognitive outcomes between the patient and control groups. On postoperative day 7 (post 7d), patients exhibited significant declines in all cognitive domains compared to HCs. Although cognitive function exhibited partial recovery by postoperative day 30 (post 30d), significant deficits remained in the Corsi block test (*P* = 0.020) and the pegboard (dominant hand) tests (*P* = 0.027). Longitudinally, after Bonferroni correction, patients exhibited significant deficits in all cognitive domains at post 7d compared to both baseline and post 30d, as assessed by the Corsi block, Digit symbol, Trail-Making Test A (TMT-A), pegboard (dominant hand), verbal fluency, and paired association learning tests. In contrast, the HCs showed no significant differences at any of the 3 time points.

**Table 2.** Results of longitudinal neuropsychological assessments of healthy controls and patients over time. Data are presented as mean ± standard deviation or median [25th and 75th percentiles]. *P*_patient_ represents *P* values among patients undergoing cardiac surgery. *P*_control_ represents *P* values among healthy controls. *P*_between-group_ represents *P* values between cardiac surgery patients and healthy controls.

Assessments	Patients	*P*_patient_	Healthy controls	*P*_control_	*P*_between-group_
	(*n* = 25)		(*n* = 25)		
Corsi block test		0.001 ^a^^,^^b^		0.272	
Baseline	47.1 ± 6.2		48.5 ± 8.6		0.503
Post 7d	39.4 ± 6.2		49.8 ± 8.3		<0.001
Post 30d	44.6 ± 5.4		49.4 ± 8.4		0.020
Digit symbol test		<0.001 ^a^^,^^b^		0.197	
Baseline	29 [24, 34]		28 [24.5, 32.5]		0.386
Post 7d	18 [10.5, 19.5]		28 [23, 31.5]		<0.001
Post 30d	28 [20, 29]		28 [24, 33]		0.089
Trail-Making Test A ^c^		<0.001 ^a^^,^^b^		0.779	
Baseline	52 [39, 57]		52 [40, 59]		0.307
Post 7d	64 [55, 72]		52 [40, 58]		<0.001
Post 30d	55 [46, 62.5]		52 [41, 58]		0.113
Pegboard (favored hand) test ^c^		<0.001 ^a^^,^^b^		0.319	
Baseline	89 [77, 109]		94 [73.5, 111]		0.900
Post 7d	111 [95, 120]		98 [70, 106.5]		<0.001
Post 30d	101 [86, 110]		94 [74, 103]		0.027
Verbal fluency test		<0.001 ^a^^,^^b^		0.627	
Baseline	37 [30.5, 39.5]		37 [30.5, 40]		0.930
Post 7d	32 [27, 37.5]		37 [32, 41]		0.014
Post 30d	34 [30, 40]		37 [32.5, 41]		0.239
Paired association learning test		<0.001 ^a^^,^^b^		0.944	
Baseline	20 [17, 20]		18 [16.5, 19]		0.129
Post 7d	17 [15, 18]		18 [16.5, 20]		0.009
Post 30d	18 [16, 19]		18 [16.5, 19.5]		0.196

Post 7d, postoperative day 7; Post 30d, postoperative day 30

^a^ Post hoc comparisons showed significant differences between baseline and post 7d.

^b^ Post hoc comparisons showed significant differences between post 7d and 30.

^c^ Lower scores indicate better performance.

### Postoperative gLFC connectivity decline and recovery in cardiac surgery patients

**Fig. 1.**
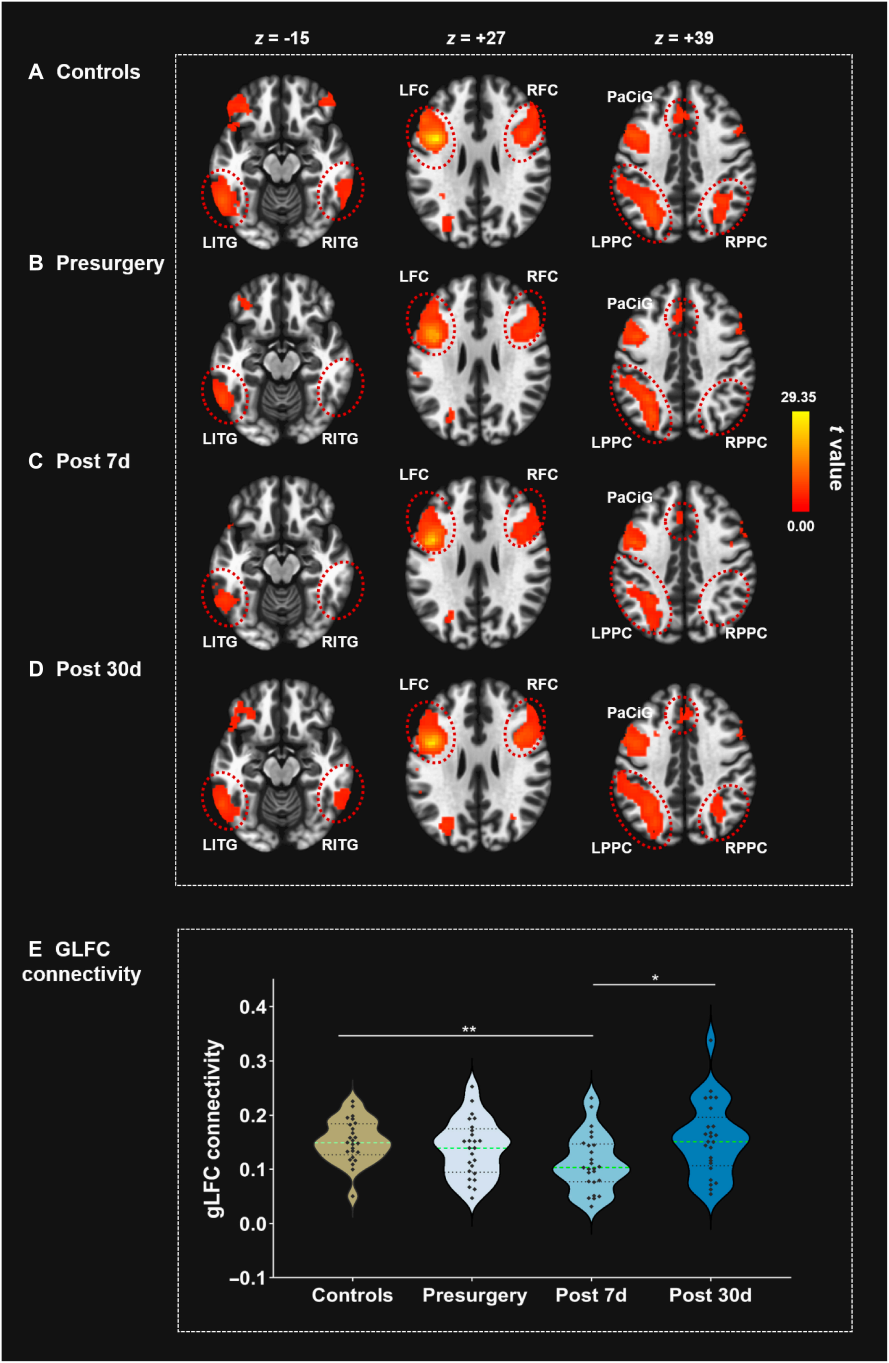
Positive functional connectivity maps of the LFC in the healthy controls and cardiac surgery patients over time. (A) Healthy controls at baseline; (B) patients at presurgery; (C) patients at postoperative day 7; (D) patients at postoperative day 30; (E) comparisons of mean gLFC connectivity between healthy controls and patients. Results were reported using an uncorrected voxel-wise height threshold of *P* < 0.001 combined with an FWE-corrected cluster-wise threshold of *P* < 0.05. The violin plot showed the average value of gLFC connectivity. LFC, left frontal cortex; RFC, right frontal cortex; LPPC, left posterior parietal cortex; RPPC, right posterior parietal cortex; LITG, left inferior temporal gyrus; RITG, right inferior temporal gyrus; PaCiG, paracingulate gyrus; Controls, healthy controls; Post 7d, postoperative day 7; Post 30d, postoperative day 30; FWE, family-wise error; gLFC connectivity, global left frontal cortex connectivity.

The positive functional connectivity patterns observed in the LFC are illustrated in Fig. 1 and Table S1. Patients exhibited a significant decline in gLFC connectivity compared to HCs (*P* = 0.004) at post 7d. At both baseline and the 30-day postoperative follow-up, gLFC connectivity values of patients were comparable to those in HCs (*P* > 0.05). Within the patient group, there were significant changes in gLFC connectivity across the 3 time points (*P* = 0.016). After Bonferroni correction, gLFC connectivity still significantly improved from post 7d to 30 in the patients (*P* = 0.039).

### Moderation analysis results

Generalized linear model (GLM) analysis showed that ΔIL-6_(7d-baseline)_ was only associated with the ΔCorsi block test_(30d-7d)_ (*β* = −0.62, 95% CI: [−0.95, −0.27], *P* = 0.001) and ΔDigit symbol test_(30d-7d)_ (*β* = −0.47, 95% CI: [−0.85, −0.09], *P* = 0.017). No significant relationship was found between changes in TNF-α levels and neuropsychological assessments (*P* > 0.05). Details were available in Table S2.

**Fig. 2.**
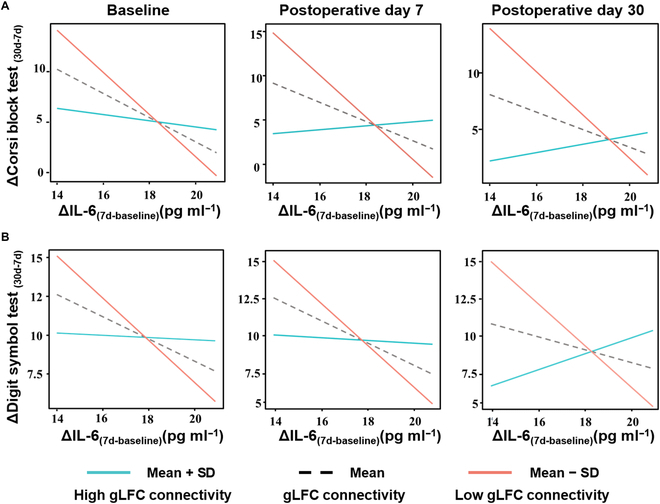
Illustration of the interaction effects of gLFC connectivity at baseline, postoperative day 7, and postoperative day 30 with ΔIL-6_(7d-baseline)_ on cognitive recovery after surgery. (A) ΔCorsi block test_(30d-7d)_. (B) ΔDigit symbol test_(30d-7d)_. Scatterplots depict the interaction between patients’ gLFC connectivity values and systemic inflammation on the recovery of cognitive performance after cardiac surgery. ΔIL-6_(7d-baseline)_, change in interleukin-6 from baseline to postoperative day 7; ΔCorsi block test_(30d-7d)_, change in Corsi test score from postoperative day 7 to 30; ΔDigit symbol test_(30d-7d)_, change in digit symbol test score from postoperative day 7 to 30; gLFC connectivity, global left frontal cortex connectivity; SD, standard deviation.

Moderation analysis showed that the interactions of gLFC connectivity at all 3 time points (baseline, post 7d, and post 30d) × ΔIL-6_(7d-baseline)_ were separately significant for the ΔCorsi block test_(30d-7d)_ and ΔDigit symbol test_(30d-7d)_, as shown in Table 3. Specifically, the interaction terms were significant for both the ΔCorsi block test (baseline: *β* = 0.39, *P* = 0.010; post 7d: *β* = 0.56, *P* = 0.001; post 30d: *β* = 0.45, *P* = 0.020) and ΔDigit symbol test (baseline: *β* = 0.40, *P* = 0.020; post 7d: *β* = 0.45, *P* = 0.035; post 30d: *β* = 0.53, *P* = 0.014). Interaction plots indicated that the negative effects of ΔIL-6_(7d-baseline)_ on cognitive outcomes were less pronounced at higher levels of gLFC connectivity at all 3 time points (Fig. 2).

**Table 3.** Moderation effects of change of IL-6 from baseline to postoperative day 7 (predictor) and gLFC connectivity (mediator) on cognitive recovery from postoperative day 7 to 30 (dependent variable). ΔIL-6_(7d-baseline)_, independent variable; postoperative cognitive performance, dependent variable; gLFC connectivity at presurgery, postoperative day 7, and postoperative day 30 separately, moderator.

	ΔCorsi block test_(30d-7d)_			ΔDigit symbol test_(30d-7d)_		
Moderator	*β* (95% CI)	*P*	Overall *R*^2^	*β* (95% CI)	*P*	Overall *R*^2^
gLFC connectivity at presurgery						
ΔIL-6_(7d-baseline)_ × gLFC connectivity	0.39 (0.10, 0.67)	0.010	0.487	0.40 (0.07, 0.72)	0.020	0.321
ΔIL-6_(7d-baseline)_	−0.54 (−0.85, −0.23)	0.002		−0.41 (−0.77, −0.06)	0.026	
gLFC connectivity	−0.04 (−0.35, 0.27)	0.795		0.05 (−0.31, 0.40)	0.783	
gLFC connectivity at postoperative day 7						
ΔIL-6_(7d-baseline)_ × gLFC connectivity	0.56 (0.24, 0.87)	0.001	0.575	0.45 (0.04, 0.86)	0.035	0.286
ΔIL-6_(7d-baseline)_	−0.49 (−0.79, −0.19)	0.003		−0.41 (−0.79, −0.02)	0.040	
gLFC connectivity	0.04 (−0.27, 0.35)	0.785		0.12 (−0.28, 0.53)	0.525	
gLFC connectivity at postoperative day 30						
ΔIL-6_(7d-baseline)_ × gLFC connectivity	0.45 (0.08, 0.82)	0.020	0.472	0.53 (0.12, 0.95)	0.014	0.344
ΔIL-6_(7d-baseline)_	−0.38 (−0.74, −0.03)	0.037		−0.26 (−0.66, 0.14)	0.188	
gLFC connectivity	−0.22 (−0.55, 0.10)	0.165		−0.002 (−0.36, 0.36)	0.989	

ΔIL-6_(7d-baseline)_, change in interleukin-6 from baseline to postoperative day 7; ΔCorsi block test_(30d-7d)_, change in Corsi block test score from postoperative day 7 to 30; ΔDigit symbol test_(30d-7d)_, change in Digit symbol test score from postoperative day 7 to 30; CI, confidence interval; gLFC connectivity, global left frontal cortex connectivity

**Fig. 3.**
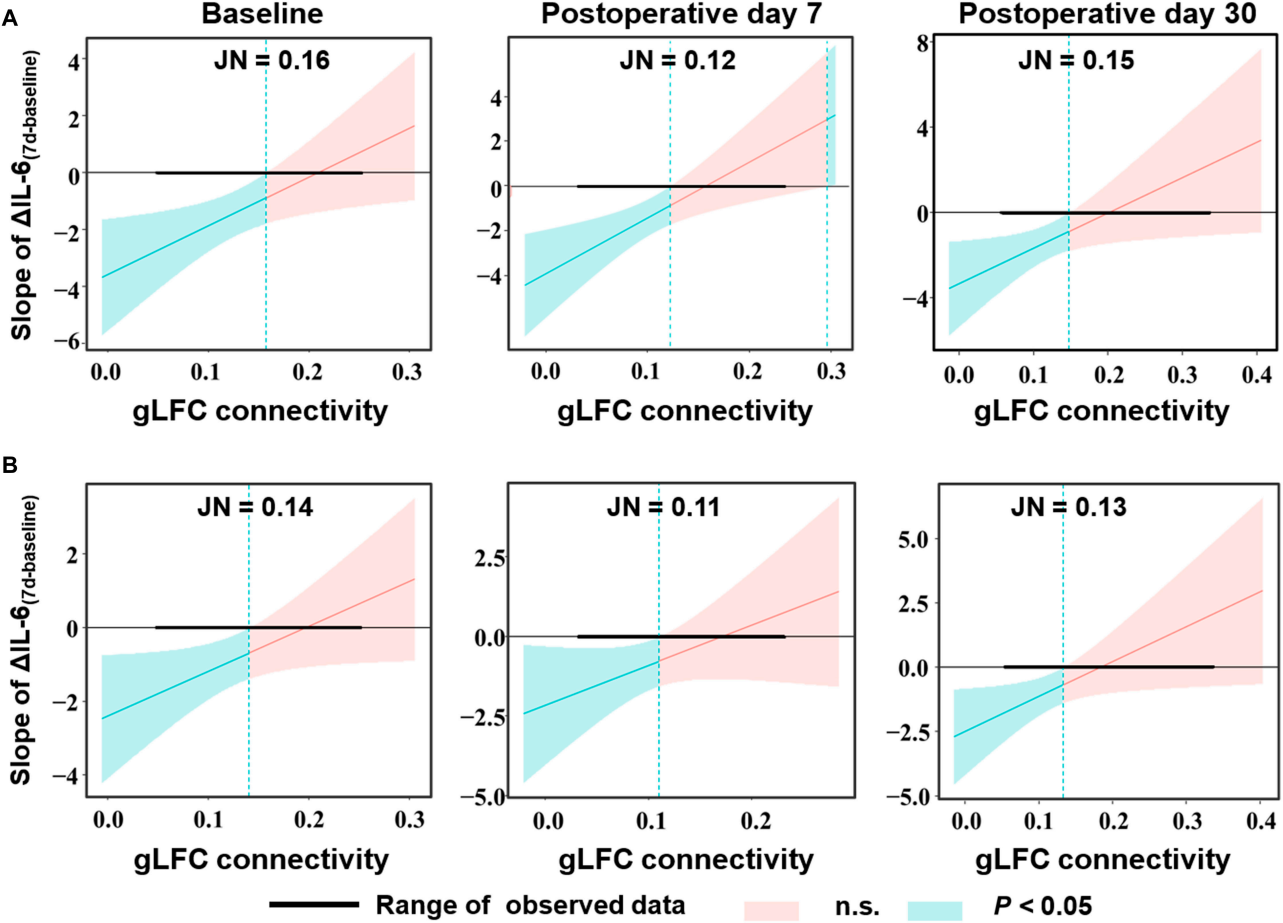
Johnson–Neyman results of gLFC connectivity. (A) ΔCorsi block test_(30d-7d)_. (B) ΔDigit symbol test_(30d-7d)_. Graphs showing the conditional effect of ΔIL-6_(7d-baseline)_ (*X*) on the change in neuropsychological assessment score from postoperative day 7 to 30 (*Y*) as a linear function of gLFC connectivity at baseline and postoperative day 7 and 30 (*W*) including the JN transition point (i.e., where the confidence interval around the condition effect crosses zero on the *y*-axis). ΔIL-6_(7d-baseline)_, change in interleukin-6 from baseline to postoperative day 7; ΔCorsi block test_(30d-7d)_, change in Corsi test score from postoperative day 7 to 30; ΔDigit symbol test_(30d-7d)_, change in digit symbol test score from postoperative day 7 to 30; gLFC connectivity, global left frontal cortex connectivity; JN, Johnson–Neyman.

Figure 3 presents the Johnson–Neyman plots, which showed the range of gLFC connectivity at 3 time points where the effect of ΔIL-6_(7d-baseline)_ on the ΔCorsi block test_(30d-7d)_ and ΔDigit symbol test_(30d-7d)_ was statistically significant. For the ΔCorsi block test_(30d-7d)_, significant interactions were observed within the gLFC connectivity ranges of [0 to 0.16] at baseline, [0 to 0.12] at post 7d, and [0 to 0.15] at post 30d. Similarly, for the ΔDigit symbol test_(30d-7d)_, significant interactions were noted within the gLFC connectivity ranges of [0 to 0.14] at baseline, [0 to 0.11] at post 7d, and [0 to 0.13] at post 30d. Within these ranges, the higher the gLFC connectivity, the less negative the effect of ΔIL-6_(7d-baseline)_ on changes in postoperative cognitive outcome, suggesting that gLFC connectivity plays a protective role against inflammation-related cognitive function. All analyses were repeated for the control regions (RFC, OP, and M1). As expected, no significant effects were detected in these 3 control regions. Detailed results are provided in Tables S3 to S5.

## Discussion

This study examined the dynamic changes in gLFC connectivity in patients following valve replacement surgery. We then explored the association between these changes, inflammatory factors, and cognitive performance. Our results suggest that higher gLFC connectivity may help protect postoperative cognition related to inflammatory factors in patients.

Existing research has mainly examined the relationship between CR and PND development, frequently utilizing static proxies reflective of early-life exposures [14]. However, they were limited in their exploration of the dynamic changes underlying CR and the underlying neurobiological mechanisms. The present study evaluated the gLFC connectivity of patients who underwent cardiac surgery, revealing a novel finding of the functional neural mechanism promoting postsurgery cognitive recovery.

Consistent with previous studies [9,22], we found that patients exhibited significant cognitive decline within the first 7 days after cardiac surgery. This decline affected memory, attention, executive function, and learning, all of which returned to near-baseline levels within 30 days after surgery. Furthermore, patients demonstrated dynamic changes in gLFC connectivity, like the change in cognitive function, suggesting adaptation to brain injury during the perioperative period. The dynamic pattern of gLFC connectivity, with decline at 7 days and recovery to preoperative levels by 30 days, closely aligned with the cognitive performance trajectories in these surgical patients [17]. Based on this finding, we hypothesized that gLFC connectivity may be the key neural substrate for promoting postoperative cognitive recovery. This is because the LFC, a key region in the frontoparietal control network [23,24], is critical for brain network resilience to targeted attacks [19,25]. It is particularly important to regulate the activity of various functional networks [26,27]. This may reflect the underlying neuroplasticity mechanisms that modulate brain function [28]. Surgery-induced modifications in gLFC connectivity underscore the brain’s inherent ability to dynamically reconfigure neural circuits in response to perioperative insults. This finding supports the notion of dynamic maintenance and preservation of brain function in the CR theory, indicating that CR is a static feature of brain structure, apart from that which can be optimized through continuous neuroplasticity to alleviate surgery-induced cognitive dysfunction [17,29].

Consistent with a previous study [30], our findings also indicated that ΔIL-6_(7d-baseline)_ was significantly negatively associated with cognitive recovery between postoperative 7 and 30 days, suggesting that the systemic inflammatory response may be associated with impaired cognitive rehabilitation after surgery. Surgical trauma activates immune pathways through damage-associated molecular pattern secretion, leading to a postoperative systemic inflammatory response characterized by elevated cerebrospinal fluid cytokine levels and microglial activation [31,32]. These higher concentrations of inflammatory markers potentially disrupt the integrity of the blood–brain barrier [7,33]. This disruption can result in enhanced trans-signaling within the hippocampal CA1 neurons. In turn, this can further increase the release of inflammatory cytokines and contribute to postoperative cognitive dysfunction [34].

Importantly, our results suggest that gLFC connectivity plays a crucial role in modulating the association between ΔIL-6_(7d-baseline)_ and postoperative cognitive recovery. Consistent with previous studies [18,19], gLFC connectivity in our patients protected cognitive function from the negative impacts of cardiac surgery. Higher CR attenuates the adverse effects of surgery-induced systemic inflammation. If the patient exhibits a high CR at either 7 or 30 days postsurgery, the early recovery of cognitive function may not be affected, even in the presence of elevated inflammatory factors. This resilience can be attributed to the brain’s capacity to harness existing cognitive resources more efficiently or to employ compensatory mechanisms to mitigate the detrimental impact of heightened inflammation on cognitive processes. The Johnson–Neyman analysis showed that gLFC connectivity moderates the negative impact of IL-6 within a certain range. Consistent with previous studies [35], enhancing gLFC connectivity is more beneficial for patients with lower levels due to a potential ceiling effect.

Although this study was strengthened using longitudinal data, it had some limitations. First, although the sample size of 25 patients and 25 HCs met the recommended minimum of 20 participants to ensure functional magnetic resonance imaging (fMRI) reliability [36], future studies should include a larger number of participants to increase the generalizability of the findings. Second, there was no statistically significant difference in the patients’ cognitive scores on post 30d compared with baseline, but certain cognitive subdomains (e.g., attention function) did not fully recover within 30 days of cardiac surgery, suggesting that extended longitudinal studies are necessary to delineate the neural mechanisms of cognitive recovery in postsurgical patients.

In conclusion, this study demonstrated that higher gLFC connectivity is associated with resistance to the negative association between inflammatory factors and postoperative cognitive recovery. These findings offer preliminary insight into how a higher CR might contribute to cognitive rehabilitation after cardiac surgery. Moreover, this study suggests that enhancement of gLFC connectivity during the perioperative period, involving cognitive training and/or transcranial magnetic stimulation, can facilitate postoperative cognitive rehabilitation, particularly in patients with lower CR per se.

## Materials and Methods

### Participants

This cohort study was approved by the Xuzhou Central Hospital IRB (Protocol Code: XZXY-LJ20170818) and registered in the Chinese Clinical Trial Registry (ChiCTR-OOC-17012542). The protocol complied with the Declaration of Helsinki, and written informed consent was obtained from all participants prior to enrollment. Patients scheduled for valve replacement surgery using cardiopulmonary bypass (CPB) and general anesthesia were evaluated for eligibility. Inclusion criteria were as follows: Eligible participants were aged 40 to 70 years, had completed at least 6 years of education, and had a Mini-Mental State Examination (MMSE) score of at least 23. Exclusion criteria were as follows: Participants were excluded if they had a history of craniocerebral surgery, cerebrovascular disease, hepatorenal failure, chronic inflammatory conditions, psychiatric disorders, alcoholism, illiteracy, left-handedness, or MRI-incompatible metal implants. Finally, there were 42 patients and 28 HCs enrolled. Following exclusions due to follow-up losses, excessive head movement during scanning, and the presence of cortical infarction or microinfarction, the final sample comprised 25 patients (14 women; mean age: 52.0 ± 10.7 years) and 25 age- and sex-matched HCs (15 women; mean age: 52.1 ± 10.0 years).

### Perioperative management

All patients received standardized general anesthesia. Induction was achieved using sequential administration of midazolam (0.05 mg/kg), cisatracurium (0.3 mg/kg), etomidate (0.3 mg/kg), and sufentanil (5 μg/kg). Anesthesia maintenance included remifentanil, sevoflurane, and propofol, with bispectral index (BIS) values maintained within the range of 40 to 60. Continuous hemodynamic monitoring encompassed heart rate, arterial blood pressure, respiratory rate, core temperature, end-tidal carbon dioxide (PETCO₂), and oxygen saturation (SpO₂). CPB was performed under mild hypothermia (32 °C) via nasopharyngeal and rectal temperature probes. Norepinephrine titration-maintained perfusion pressure was at 60 to 80 mmHg, while pump flow rates were kept at 2.0 to 2.5 L/min/m^2^. Hematocrit levels were strictly controlled (>21% during CPB; >25% perioperatively) with intraoperative blood salvage. Temperature management included α-stat pH regulation and controlled rewarming at 0.25 °C/min. Analgesia Protocol: Phase 1: Target-controlled infusion of hydromorphone (plasma concentration 2 ng/ml) from intensive care unit admission until extubation. Phase 2: Patient-controlled analgesia via the CP-E200 system (Zhejiang Sujia Medical Device Co., Ltd., Jiaxing, China), delivering 0.3 μg/kg/min continuous infusion with optional 0.5 mg bolus hourly, sustained for the first 48 postoperative hours.

### Clinical assessments

Neuropsychological assessments were conducted to assess cognitive function in patients at 3 time points: preoperatively (baseline), post 7d, and post 30d. HCs were conducted at the same time intervals. The assessments included the Corsi block test for memory, Digit symbol test and TMT-A for attention, pegboard (favorable hand) and verbal fluency tests for executive function, and paired associative verbal learning test for learning ability. Postoperative cognitive recovery was measured as the changes in neuropsychological assessment scores between post 7d and post 30d (ΔNeuropsychological assessment_[30d–7d]_).

Blood samples were collected at all 3 time points for patients, while HCs were sampled only once. All samples were uniformly obtained at about 8:00 AM under fasting conditions to minimize the influence of diurnal variation on inflammatory cytokine levels. At baseline, no patients received anti-inflammatory medications. Postoperative pharmacological management was standardized across all patients to control for potential confounding effects on inflammatory markers. The levels of IL-6 and TNF-α were quantified using enzyme-linked immunosorbent assay kits (ABclonal Biotechnology Co., Ltd., Woburn, MA, USA). The change in inflammatory markers (ΔInflammatory factor_[7d–baseline]_) was calculated to assess the inflammatory response following cardiac surgery.

### MRI data acquisition

Imaging was performed using a Siemens Skyra 3 Tesla scanner equipped with a 20-channel head coil (Siemens, Erlangen, Germany). During scanning, participants were instructed to maintain their eyes closed to minimize movement artifacts. Cardiac surgery patients underwent resting-state fMRI (rs-fMRI) at 3 time points: baseline, post 7d, and post 30d. HCs were scanned once at baseline for comparative analysis. The imaging protocol included 3 sequences: (a) Rs-fMRI: repetition time (TR) = 2,000 ms, echo time (TE) = 30 ms, flip angle (FA) = 90°, acquisition matrix = 64 × 64, field of view (FOV) = 220 mm × 220 mm, voxel size = 3.44 × 3.44 × 3 mm^3^, 35 slices, and 210 time points; (b) Three-dimensional T1-weighted magnetization-prepared rapid gradient echo: 192 sagittal slices, 1 × 1 × 1 mm^3^ voxel size, TR = 2,530 ms, TE = 2.98 ms, FA = 7°, acquisition matrix = 256 × 256, and FOV = 256 mm × 224 mm; and (c) Axial T2-weighted imaging: 18 slices, slice thickness = 6 mm, TR = 6,000 ms, TE = 99 ms, acquisition matrix = 320 × 320, and FOV = 230 mm × 230 mm.

### MRI data preprocessing

Rs-fMRI data processing was carried out using MATLAB DPABI v7 [37] and CONN v22a [38]. To stabilize signal fluctuations, the initial 10 functional volumes were removed, leaving 200 images for further analysis. Subsequent preprocessing steps involved correcting for slice-timing differences and realigning images to reduce head motion artifacts. Participants with excessive motion (>3.0 mm translation or >3° rotation) were excluded. The adjusted images were then co-registered with T1-weighted anatomical scans and normalized to the MNI template using segmentation-based transformation, with resampling to a voxel dimension of 3 × 3 × 3 mm^3^. Spatial smoothing was achieved with a 6-mm full-width at half-maximum isotropic Gaussian kernel, while temporal filtering (0.008 to 0.09 Hz) was applied to suppress physiological noise. Artifact detection procedures confirmed that over 90% of the scans remained valid. Finally, denoising was performed by regressing out 6 motion parameters along with their derivatives, applying CompCor-based regression for white matter and cerebrospinal fluid signals [39], and incorporating linear detrending to enhance data robustness for subsequent analyses.

### Assessment of gLFC connectivity

According to a previous study [24], gLFC connectivity was assessed using seed-to-voxel connectivity analysis in the CONN toolbox. The left frontal cortex (LFC) region of interest (ROI) was defined at MNI coordinates (−42, +6, +28) with an 8-mm radius. Fisher’s *r*-to-*z*-transformed Pearson correlation coefficients were computed between the LFC time series and gray matter voxels. Voxel-wise GLMs were applied to global LFC maps for whole-brain comparisons. Results were reported with an uncorrected threshold of *P* < 0.001 and an FWE-corrected cluster threshold of *P* < 0.05. Positive correlations were averaged across time points to derive gLFC connectivity values. To assess the regional specificity of the gLFC findings, connectivity was additionally examined in 3 control ROIs. These comprised the following: (a) the right frontal cortex (RFC; +42, +6, +28), serving as the homotopic region to evaluate hemispheric lateralization; and (b) 2 functionally distinct regions: the occipital pole (OP; –19, –102, –3) and the primary motor cortex (M1; –38, –22, +56). Group-level rather than subject-specific ROIs were employed for these analyses due to the lack of an established standard for defining individualized functional seed regions.

### Statistical analysis

The sample size was determined based on data availability within a hypothesis-driven study design. Given that our previous study demonstrated fMRI connectivity differences between 17 patients and 18 controls [9], we anticipate that our sample size is sufficient to identify factors influencing cognitive performance in patients undergoing cardiac surgery.

Continuous variables were analyzed as mean ± standard deviation. Between-group comparisons (patients vs. HCs) employed independent *t* tests, while within-group temporal comparisons utilized repeated-measures analysis of variance (ANOVA) with Bonferroni correction. Nonnormal continuous data are presented as median (interquartile range) and analyzed using nonparametric tests for intergroup comparisons and Friedman tests with post hoc adjustments for intragroup comparisons. Categorical variables are expressed as *n* (%), with intergroup differences assessed via *χ*^2^ tests.

To observe the impact of cardiac surgery on patients’ gLFC connectivity, we extracted gLFC connectivity values from fMRI data at 3 time points for the patient group: baseline, post 7d, and post 30d. We also extracted the HCs’ gLFC connectivity values at baseline. We first conducted a 2-sample *t* test to examine intergroup differences. A Bonferroni-adjusted ANOVA was conducted to assess gLFC connectivity among cardiac surgery patients across 3 time points.

To comprehensively explore the relationship among gLFC connectivity, inflammatory factors, and cognitive function, we initially performed a linear regression analysis (GLM) to investigate the association between changes in inflammatory markers and cognitive recovery outcomes. The independent variable was the change in inflammatory factors (Δinflammatory factor_[7d-baseline]_). The dependent variable was the change in neuropsychological assessments (ΔNeuropsychological assessment_[30d-7d]_). Further analysis of the moderating effects was conducted based on the significance of the results of the GLM. We investigated the separate moderating effects of patients’ gLFC connectivity at baseline, post 7d, and post 30d on the impact of cardiac surgery. All the variables were transformed into *z*-scores. Then, we conducted Johnson–Neyman analyses to identify significant boundaries for the moderator [40]. Analyses were conducted using R version 4.3.3 (R Foundation for Statistical Computing, 2023) and SPSS version 26, with a significance level of *α* = 0.05.

## Data Availability

Please contact the corresponding authors for data requests.

## References

[1] Moller J, Cluitmans P, Rasmussen L, Houx P, Rasmussen H, Canet J, Rabbitt P, Jolles J, Larsen K, Hanning C, et al. Long-term postoperative cognitive dysfunction in the elderly: ISPOCD1 study. Lancet. 1998;351(9106):857–861. 9525362 10.1016/s0140-6736(97)07382-0

[2] Evered L, Silbert B, Knopman DS, Scott DA, DeKosky ST, Rasmussen LS, Oh ES, Crosby G, Berger M, Eckenhoff RG, et al. Recommendations for the nomenclature of cognitive change associated with anaesthesia and surgery—2018. J Alzheimers Dis. 2018;66(1):1–10. 30347621 10.3233/JAD-189004

[3] Steinmetz J, Christensen KB, Lund T, Lohse N, Rasmussen LS, ISPOCD Group. Long-term consequences of postoperative cognitive dysfunction. Anesthesiology. 2009;110(3):548–555. 19225398 10.1097/ALN.0b013e318195b569

[4] Price CC, Garvan CW, Monk TG. Type and severity of cognitive decline in older adults after noncardiac surgery. Anesthesiology. 2008;108(1):8–17. 18156877 10.1097/01.anes.0000296072.02527.18 PMC2911011

[5] Bhushan S, Li Y, Huang X, Cheng H, Gao K, Xiao Z. Progress of research in postoperative cognitive dysfunction in cardiac surgery patients: A review article. Int J Surg. 2021;95:106163. 34743049 10.1016/j.ijsu.2021.106163

[6] Bowyer AJ, Heiberg J, Sessler DI, Newman S, Royse AG, Royse CF. Validation of the cognitive recovery assessments with the postoperative quality of recovery scale in patients with low-baseline cognition. Anaesthesia. 2018;73(11):1382–1391. 30084176 10.1111/anae.14402

[7] Alam A, Hana Z, Jin Z, Suen KC, Ma D. Surgery, neuroinflammation and cognitive impairment. EBioMedicine. 2018;37:547–556. 30348620 10.1016/j.ebiom.2018.10.021 PMC6284418

[8] Subramaniyan S, Terrando N. Neuroinflammation and perioperative neurocognitive disorders. Anesth Analg. 2019;128(4):781–788. 30883423 10.1213/ANE.0000000000004053 PMC6437083

[9] Zhu Y, Zhou M, Jia X, Zhang W, Shi Y, Bai S, Rampes S, Vizcaychipi MP, Wu C, Wang K, et al. Inflammation disrupts the brain network of executive function after cardiac surgery. Ann Surg. 2023;277(3):e689–e698. 34225294 10.1097/SLA.0000000000005041 PMC9891271

[10] Stern Y. What is cognitive reserve? Theory and research application of the reserve concept. J Int Neuropsychol Soc. 2002;8(3):448–460. 11939702

[11] Stern Y. Cognitive reserve in ageing and Alzheimer’s disease. Lancet Neurol. 2012;11(11):1006–1012. 23079557 10.1016/S1474-4422(12)70191-6 PMC3507991

[12] Humeidan ML, Reyes J-PC, Mavarez-Martinez A, Roeth C, Nguyen CM, Sheridan E, Zuleta-Alarcon A, Otey A, Abdel-Rasoul M, Bergese SD. Effect of cognitive prehabilitation on the incidence of postoperative delirium among older adults undergoing major noncardiac surgery. JAMA Surg. 2020;156(2):148–156. 10.1001/jamasurg.2020.4371 PMC7658803 33175114

[13] Jiang Y, Xie Y, Fang P, Shang Z, Chen L, Zhou J, Yang C, Zhu W, Hao X, Ding J, et al. Cognitive training for reduction of delirium in patients undergoing cardiac surgery. JAMA Netw Open. 2024;7(4): Article e247361. 38652478 10.1001/jamanetworkopen.2024.7361 PMC11040409

[14] Megari K, Kosmidis MH. Protecting the brain while healing hearts: The protective role of cognitive reserve in cardiac surgery. Am J Geriatr Psychiatry. 2023;32(2):195–204. 37926673 10.1016/j.jagp.2023.10.003

[15] Soldan A, Pettigrew C, Cai Q, Wang J, Wang M-C, Moghekar A, Miller MI, Albert M. Cognitive reserve and long-term change in cognition in aging and preclinical Alzheimer’s disease. Neurobiol Aging. 2017;60:164–172. 28968586 10.1016/j.neurobiolaging.2017.09.002 PMC5679465

[16] Wilson RS, Yu L, Lamar M, Schneider JA, Boyle PA, Bennett DA. Education and cognitive reserve in old age. Neurology. 2019;92(10):e1041–e1050. 30728309 10.1212/WNL.0000000000007036 PMC6442015

[17] Bettcher BM, Gross AL, Gavett BE, Widaman KF, Fletcher E, Dowling NM, Buckley RF, Arenaza-Urquijo EM, Zahodne LB, Hohman TJ, et al. Dynamic change of cognitive reserve: Associations with changes in brain, cognition, and diagnosis. Neurobiol Aging. 2019;83:95–104. 31585371 10.1016/j.neurobiolaging.2019.08.016 PMC6977973

[18] Neitzel J, Franzmeier N, Rubinski A, Ewers M. Initiative for the ADN. Left frontal connectivity attenuates the adverse effect of entorhinal tau pathology on memory. Neurology. 2019;93(4):e347–e357. 31235661 10.1212/WNL.0000000000007822 PMC6669934

[19] Franzmeier N, Hartmann JC, Taylor ANW, Araque Caballero MÁ, Simon-Vermot L, Buerger K, Kambeitz-Ilankovic LM, Ertl-Wagner B, Mueller C, Catak C, et al. Left frontal hub connectivity during memory performance supports reserve in aging and mild cognitive impairment. J Alzheimers Dis. 2017;59(4):1381–1392. 28731448 10.3233/JAD-170360 PMC5611800

[20] Franzmeier N, Düzel E, Jessen F, Buerger K, Levin J, Duering M, Dichgans M, Haass C, Suárez-Calvet M, Fagan AM, et al. Left frontal hub connectivity delays cognitive impairment in autosomal-dominant and sporadic Alzheimer’s disease. Brain. 2018;141(4):1186–1200. 29462334 10.1093/brain/awy008 PMC5888938

[21] Franzmeier N, Hartmann J, Taylor ANW, Araque-Caballero MÁ, Simon-Vermot L, Kambeitz-Ilankovic L, Bürger K, Catak C, Janowitz D, Müller C, et al. The left frontal cortex supports reserve in aging by enhancing functional network efficiency. Alzheimers Res Ther. 2018;10(1):28. 29510747 10.1186/s13195-018-0358-y PMC5838935

[22] Relander K, Hietanen M, Rantanen K, Rämö J, Vento A, Saastamoinen K, Roine RO, Soinne L. Postoperative cognitive change after cardiac surgery predicts long-term cognitive outcome. Brain Behav. 2020;10: Article e01750. 32681544 10.1002/brb3.1750 PMC7507551

[23] Cole MW, Pathak S, Schneider W. Identifying the brain’s most globally connected regions. NeuroImage. 2010;49(4):3132–3148. 19909818 10.1016/j.neuroimage.2009.11.001

[24] Cole MW, Yarkoni T, Repovs G, Anticevic A, Braver TS. Global connectivity of prefrontal cortex predicts cognitive control and intelligence. J Neurosci. 2012;32(26):8988–8999. 22745498 10.1523/JNEUROSCI.0536-12.2012 PMC3392686

[25] Cole MW, Repov G, Anticevic A. The frontoparietal control system: A central role in mental health. Neuroscientist. 2014;20(6):652–664. 24622818 10.1177/1073858414525995 PMC4162869

[26] Cole MW, Reynolds JR, Power JD, Repovs G, Anticevic A, Braver TS. Multi-task connectivity reveals flexible hubs for adaptive task control. Nat Neurosci. 2013;16(9):1348–1355. 23892552 10.1038/nn.3470 PMC3758404

[27] Varela-López B, Cruz-Gómez ÁJ, Lojo-Seoane C, Díaz F, Pereiro AX, Zurrón M, Lindín M, Galdo-Álvarez S. Cognitive reserve, neurocognitive performance, and high-order resting-state networks in cognitively unimpaired aging. Neurobiol Aging. 2022;117:151–164. 35759984 10.1016/j.neurobiolaging.2022.05.012

[28] Park DC, Bischof GN. The aging mind: Neuroplasticity in response to cognitive training. Dialogues Clin Neurosci. 2013;15(1):109–119. 23576894 10.31887/DCNS.2013.15.1/dpark PMC3622463

[29] Cabeza R, Albert M, Belleville S, Craik FIM, Duarte A, Grady CL, Lindenberger U, Nyberg L, Park DC, Reuter-Lorenz PA, et al. Maintenance, reserve and compensation: The cognitive neuroscience of healthy ageing. Nat Rev Neurosci. 2018;19(11):701–710. 30305711 10.1038/s41583-018-0068-2 PMC6472256

[30] Taylor J, Wu JG, Kunkel D, Parker M, Rivera C, Casey C, Naismith S, Teixeira-Pinto A, Maze M, Pearce RA, et al. Resolution of elevated interleukin-6 after surgery is associated with return of normal cognitive function. Br J Anaesth. 2023;131(4):694–704. 37385855 10.1016/j.bja.2023.05.023 PMC10925892

[31] Wan Y, Xu J, Meng F, Bao Y, Ge Y, Lobo N, Vizcaychipi MP, Zhang D, Gentleman SM, Maze M, et al. Cognitive decline following major surgery is associated with gliosis, β-amyloid accumulation, and τ phosphorylation in old mice. Crit Care Med. 2010;38(11):2190–2198. 20711073 10.1097/CCM.0b013e3181f17bcb

[32] Vizcaychipi MP, Watts HR, O’Dea KP, Lloyd DG, Penn JW, Wan Y, Pac-Soo C, Takata M, Ma D. The therapeutic potential of atorvastatin in a mouse model of postoperative cognitive decline. Ann Surg. 2014;259(6):1235–1244. 24263322 10.1097/SLA.0000000000000257

[33] Yang S, Gu C, Mandeville ET, Dong Y, Esposito E, Zhang Y, Yang G, Shen Y, Fu X, Lo EH, et al. Anesthesia and surgery impair blood–brain barrier and cognitive function in mice. Front Immunol. 2017;8:902. 28848542 10.3389/fimmu.2017.00902 PMC5552714

[34] Hu J, Zhang Y, Huang C, Feng X, He S, Zhang Y, Maze M. Interleukin-6 trans-signalling in hippocampal CA1 neurones mediates perioperative neurocognitive disorders in mice. Br J Anaesth. 2022;129(6):923–936. 36253222 10.1016/j.bja.2022.08.019

[35] Lv T, You S, Qin R, Hu Z, Ke Z, Yao W, Zhao H, Xu Y, Bai F. Distinct reserve capacity impacts on default-mode network in response to left angular gyrus-navigated repetitive transcranial magnetic stimulation in the prodromal Alzheimer disease. Behav Brain Res. 2023;439: Article 114226. 36436729 10.1016/j.bbr.2022.114226

[36] Thirion B, Pinel P, Mériaux S, Roche A, Dehaene S, Poline J-B. Analysis of a large fMRI cohort: Statistical and methodological issues for group analyses. NeuroImage. 2007;35(1):105–120. 17239619 10.1016/j.neuroimage.2006.11.054

[37] Yan C-G, Wang X-D, Zuo X-N, Zang Y-F. DPABI: Data Processing & Analysis for (resting-state) brain imaging. Neuroinformatics. 2016;14(3):339–351. 27075850 10.1007/s12021-016-9299-4

[38] Whitfield-Gabrieli S, Nieto-Castanon A. Conn: A functional connectivity toolbox for correlated and anticorrelated brain networks. Brain Connect. 2012;2(3):125–141. 22642651 10.1089/brain.2012.0073

[39] Behzadi Y, Restom K, Liau J, Liu TT. A component based noise correction method (CompCor) for BOLD and perfusion based fMRI. NeuroImage. 2007;37(1):90–101. 17560126 10.1016/j.neuroimage.2007.04.042 PMC2214855

[40] Johnson PO, Fay LC. The Johnson-Neyman technique, its theory and application. Psychometrika. 1950;15(4):349–367. 14797902 10.1007/BF02288864

