# Supplementary material for: Left Frontal Connectivity Moderates the Relationship between Inflammation and Cognitive Performance in Patients following Cardiac Surgery: A Longitudinal fMRI Study
Source: Research (Wash D C). 2025 Sep 19;8:0893. doi: 10.34133/research.0893 (PMC12446753; doi:10.34133/research.0893)
Supplement: Supplementary 1 — Tables S1 to S5 [file research.0893.f1.docx]

SUPPLEMENTARY MATERIALS: Tables S1 to S5

**Supplementary Table 1.** Brain regions significantly positively correlated with LFC in healthy controls and cardiac surgery patients over time

| **Anatomical region** | **Cluster size (voxels)** | **MNI (*x, y, z*)** | ***t*-value** |
| --- | --- | --- | --- |
| **Controls: Baseline** |  |  |  |
| Lt. MidFG | 1782 | (-39, +06, +27) | 19.33 |
| Rt. MidFG | 601 | (+42, +12, +27) | 12.48 |
| Bi. PaCiG | 145 | (+9, +18, +48) | 6.54 |
| Lt. PPC | 909 | (-33, -54, +45) | 10.20 |
| Rt. PPC | 262 | (+33, -51, +39) | 6.88 |
| Lt. ITG | 560 | (-57, -52, -15) | 12.48 |
| Rt. ITG | 180 | (+63, -51, -18) | 7.47 |
| **Patients: Baseline** | | | |
| Lt. MidFG | 1630 | (-42, +09, +24) | 17.99 |
| Rt. MidFG | 493 | (+45, +12, +24) | 10.01 |
| Bi. PaCiG | 127 | (+00, +15, +51) | 7.73 |
| Lt. PPC | 600 | (-33, -51, +42) | 9.83 |
| Rt. PPC | 53 | (+33, -63, +48) | 7.69 |
| Lt. ITG | 286 | (-57, -57, -12) | 8.36 |
| Rt. ITG | 37 | (+66, -51, -12) | 5.62 |
| **Patients: Postoperative day 7** | | | |
| Lt. MidFG | 1409 | (-42, +06, +27) | 16.52 |
| Rt. MidFG | 511 | (+48, +12, +21) | 9.41 |
| Bi. PaCiG | 121 | (-03, +06, +54) | 7.16 |
| Lt. PPC | 393 | (-33, -45, +39) | 7.53 |
| Rt. PPC | 76 | (+30, -54, +36) | 6.57 |
| Lt. ITG | 277 | (-57, -51, -15) | 8.28 |
| **Patients: Postoperative day 30** | | | |
| Lt. MidFG | 2012 | (-42, +06, +27) | 14.20 |
| Rt. MidFG | 661 | (+45, +12, +24) | 9.62 |
| Bi. PaCiG | 145 | (+9, +18, +48) | 6.77 |
| Lt. PPC | 989 | (-33, -57, +36) | 8.77 |
| Rt. PPC | 133 | (+30, -57, +36) | 6.24 |
| Lt. ITG | 435 | (-57, -54, -15) | 8.50 |
| Rt. ITG | 112 | (+51, -48, -24) | 5.86 |

***Note:*** Results were reported using an uncorrected voxel-wise height threshold of *p* < 0.001 combined with an FWE-corrected cluster-wise threshold of *p* < 0.05.

**Abbreviations:** MidFG, middle frontal gyrus; PPC, posterior parietal cortex; ITG, inferior temporal gyrus; PaCiG, paracingulate gyrus; Controls, healthy controls; Bi, bilateral; Lt, left; Rt, right; FWE, familywise error.

**Supplementary Table 2.** General linear regression analysis examining the relationship between the change in postoperative cognition from postoperative day 7 to 30 and the change in inflammation factors from baseline to postoperative day 7

| **Independent variables** | **ΔIL-6_(7d-baseline)_** | | **ΔTNF-α_(7d-baseline)_** | |
| --- | --- | --- | --- | --- |
|  | ***β* (95 % CI)** | ***p*** | ***β* (95 % CI)** | ***p*** |
| **ΔCorsi block test_(30d-7d)_** | -0.62 (-0.95, -0.27) | 0.001 | -0.01 (-0.71, 0.67) | 0.953 |
| **ΔDigit symbol test_(30d-7d)_** | -0.47 (-0.85, -0.09) | 0.017 | 0.04 (-0.47, 0.56) | 0.853 |
| **ΔTrail-Making test A_(30d-7d)_** | -0.07 (-0.50, 0.36) | 0.749 | 0.07 (-0.46, 0.60) | 0.789 |
| **ΔPegboard (favoured hand) test_(30d-7d)_** | 0.32 (-0.09, 0.73) | 0.120 | 0.14 (-0.88, 1.73) | 0.510 |
| **ΔVerbal fluency test_(30d-7d)_** | 0.11 (-0.32, 0.54) | 0.591 | 0.12 (-0.15, 0.27) | 0.577 |
| **ΔPaired association learning test_(30d-7d)_** | 0.01 (-0.42, 0.44) | 0.952 | -0.08 (-0.23, 0.16) | 0.693 |

**Note:** Single-exposure model with no adjustment.

**Abbreviations:** ΔIL-6_(7d-baseline)_, change in interleukin-6 from baseline to postoperative day 7; ΔCorsi block test_(30d-7d)_, change in Corsi test score from postoperative day 7 to 30; ΔDigit symbol test_(30d-7d)_, change in digit symbol test score from postoperative day 7 to 30;ΔTrail-Making Test A_(30d-7d)_, change in Trail-Making Test score from postoperative day 7 to 30; ΔPegboard (favoured hand) test_(30d-7d)_, change in Pegboard (favoured hand) test score from postoperative day 7 to 30; ΔVerbal fluency test_(30d-7d)_, change in Verbal fluency test score from postoperative day 7 to 30; ΔPaired association learning _(30d-7d)_, change in Paired association learning score from postoperative day 7 to 30; CI, confidence interval; gLFC-connectivity, global left frontal cortex connectivity

**Supplementary Table 3. Summary of moderate analysis models for global RFC connectivity**

|  | **ΔCorsi block test_(30d-7d)_** | | | **ΔDigit symbol test_(30d-7d)_** | | |
| --- | --- | --- | --- | --- | --- | --- |
| **Moderator** | ***β* (95% CI)** | ***p*** | **Overall *R*^2^** | ***β* (95% CI)** | ***p*** | **Overall *R*^2^** |
| **gRFC connectivity at pre-surgery** |  |  |  |  |  |  |
| ΔIL-6_(7d-baseline)_ × gRFC connectivity | 0.18 (-0.22, 0.58) | 0.360 | 0.404 | 0.08 (-0.37, 0.53) | 0.719 | 0.230 |
| ΔIL-6_(7d-baseline)_ | -0.63 (-0.98, -0.28) | 0.001 |  | -0.47 (-0.87, -0.07) | 0.020 |  |
| gRFC connectivity | 0.04 (-0.31, 0.39) | 0.688 |  | -0.04 (-0.44, 0.36) | 0.822 |  |
| **gRFC connectivity at postoperative day 7** |  |  |  |  |  |  |
| ΔIL-6_(7d-baseline)_ × gRFC connectivity | 0.34 (-0.18, 0.86) | 0.183 | 0.428 | -0.10 (-0.68, 0.48) | 0.729 | 0.280 |
| ΔIL-6_(7d-baseline)_ | -0.56 (-0.93, -0.18) | 0.006 |  | -0.58 (-0.99, -0.15) | 0.010 |  |
| gRFC connectivity | -0.13 (-0.54, 0.28) | 0.521 |  | 0.28 (-0.18, 0.75) | 0.222 |  |
| **gRFC connectivity at postoperative day 30** |  |  |  |  |  |  |
| ΔIL-6_(7d-baseline)_ × gRFC connectivity | 0.05 (-0.12, 0.59) | 0.187 | 0.428 | 0.28 (-0.11, 0.67) | 0.155 | 0.296 |
| ΔIL-6_(7d-baseline)_ | -0.53 (-0.92, -0.14) | 0.011 |  | -0.36 (-0.80, 0.08) | 0.100 |  |
| gRFC connectivity | -0.02 (-0.40, 0.35) | 0.898 |  | -0.06 (-0.47, 0.36) | 0.778 |  |

**Note:** Single-exposure model with no adjustment.

**Abbreviations:** ΔIL-6_(7d-baseline)_, change in interleukin-6 from baseline to postoperative day 7; ΔCorsi block test_(30d-7d)_, change in Corsi block test score from postoperative day 7 to 30; ΔDigit symbol test_(30d-7d)_, change in Digit symbol test score from postoperative day 7 to 30; CI, confidence interval; gRFC connectivity, global right frontal cortex connectivity.

**Supplementary Table 4. Summary of moderate analysis models for global occipital pole connectivity**

|  | **ΔCorsi block test_(30d-7d)_** | | | **ΔDigit symbol test_(30d-7d)_** | | |
| --- | --- | --- | --- | --- | --- | --- |
| **Moderator** | ***β* (95% CI)** | ***p*** | **Overall *R*^2^** | ***β* (95% CI)** | ***p*** | **Overall *R*^2^** |
| **gOP connectivity at pre-surgery** |  |  |  |  |  |  |
| ΔIL-6_(7d-baseline)_ × gOP connectivity | 0.34 (-0.01, 0.70) | 0.057 | 0.541 | -0.36 (-0.76, 0.05) | 0.084 | 0.400 |
| ΔIL-6_(7d-baseline)_ | -0.62 (-0.93, -0.31) | < 0.001 |  | -0.54 (-0.89, -0.18) | 0.005 |  |
| gOP connectivity | -0.22 (-0.54, 0.09) | 0.160 |  | -0.35 (-0.71, 0.01) | 0.058 |  |
| **gOP connectivity at postoperative day 7** |  |  |  |  |  |  |
| ΔIL-6_(7d-baseline)_ × gOP connectivity | 0.27 (-0.09, 0.62) | 0.131 | 0.481 | -0.22 (-0.61, 0.17) | 0.255 | 0.357 |
| ΔIL-6_(7d-baseline)_ | -0.54 (-0.88, -0.20) | 0.003 |  | -0.49 (-0.86, -0.11) | 0.013 |  |
| gOP connectivity | -0.23 (-0.56, 0.10) | 0.166 |  | -0.29 (-0.66, 0.08) | 0.114 |  |
| **gOP connectivity at postoperative day 30** |  |  |  |  |  |  |
| ΔIL-6_(7d-baseline)_ × gOP connectivity | 0.36 (-0.05, 0.78) | 0.085 | 0.471 | -0.24 (-0.72, 0.24) | 0.316 | 0.301 |
| ΔIL-6_(7d-baseline)_ | -0.53 (-0.87, -0.19) | 0.004 |  | -0.50 (-0.89, -0.11) | 0.015 |  |
| gOP connectivity | -0.19 (-0.53, 0.16) | 0.271 |  | -0.16 (-0.55, 0.24) | 0.418 |  |

**Note:** Single-exposure model with no adjustment.

**Abbreviations:** ΔIL-6_(7d-baseline)_, change in interleukin-6 from baseline to postoperative day 7; ΔCorsi block test_(30d-7d)_, change in Corsi block test score from postoperative day 7 to 30; ΔDigit symbol test_(30d-7d)_, change in Digit symbol test score from postoperative day 7 to 30; CI, confidence interval; gOP connectivity, global occipital pole connectivity.

**Supplementary Table 5. Summary of moderate analysis models for global M1 connectivity**

|  | **ΔCorsi block test_(30d-7d)_** | | | **ΔDigit symbol test_(30d-7d)_** | | |
| --- | --- | --- | --- | --- | --- | --- |
| **Moderator** | ***β* (95% CI)** | ***p*** | **Overall *R*^2^** | ***β* (95% CI)** | ***p*** | **Overall *R*^2^** |
| **gM1 connectivity at pre-surgery** |  |  |  |  |  |  |
| ΔIL-6_(7d-baseline)_ × gM1 connectivity | 0.32 (-0.09, 0.73) | 0.115 | 0.448 | 0.41 (-0.04, 0.86) | 0.071 | 0.339 |
| ΔIL-6_(7d-baseline)_ | -0.65 (-0.99, -0.31) | 0.001 |  | -0.51 (-0.88, -0.14) | 0.010 |  |
| gM1 connectivity | 0.03 (-0.31, 0.37) | 0.876 |  | -0.02 (-0.39, 0.36) | 0.930 |  |
| **gM1 connectivity at postoperative day 7** |  |  |  |  |  |  |
| ΔIL-6_(7d-baseline)_ × gM1 connectivity | 0.25 (-0.17, 0.66) | 0.232 | 0.665 | 0.39 (-0.03, 0.81) | 0.067 | 0.435 |
| ΔIL-6_(7d-baseline)_ | -0.62 (-0.98, -0.26) | 0.002 |  | -0.51 (-0.87, -0.14) | 0.010 |  |
| gM1 connectivity | 0.16 (-0.20, 0.51) | 0.366 |  | 0.33 (-0.02, 0.69) | 0.060 |  |
| **gM1 connectivity at postoperative day 30** |  |  |  |  |  |  |
| ΔIL-6_(7d-baseline)_ × gM1 connectivity | 0.09 (-0.40, 0.57) | 0.719 | 0.383 | -0.30 (-0.82, 0.23) | 0.258 | 0.271 |
| ΔIL-6_(7d-baseline)_ | -0.62 (-0.98, -0.26) | 0.002 |  | -0.47 (-0.86, -0.08) | 0.020 |  |
| gM1 connectivity | 0.04 (-0.33, 0.41) | 0.819 |  | 0.07 (-0.32, 0.47) | 0.707 |  |

**Note:** Single-exposure model with no adjustment.

**Abbreviations:** ΔIL-6_(7d-baseline)_, change in interleukin-6 from baseline to postoperative day 7; ΔCorsi block test_(30d-7d)_, change in Corsi block test score from postoperative day 7 to 30; ΔDigit symbol test_(30d-7d)_, change in Digit symbol test score from postoperative day 7 to 30; CI, confidence interval; gM1 connectivity, global M1 connectivity
